# Vaccine Issues for Adult Women^1^

**DOI:** 10.3201/eid1011.040623_12

**Published:** 2004-11

**Authors:** Susan Reef, Susan Lett, Pierce Gardner

**Affiliations:** * Centers for Disease Control and Prevention, Atlanta, Georgia, USA;; † Massachusetts Department of Public Health, Boston, Massachusetts, USA;; ‡ National Institutes of Health, Bethesda, Maryland, USA

**Keywords:** adult women, immunizations, vaccine recommendations, pneumococcal disease, influenza

## Pertussis in Adolescents and Adults in Massachusetts

Pertussis, also known as whooping cough, is commonly characterized by an acute cough with either paroxysms of cough, inspiratory whoop, or post-tussive vomiting. Complications of pertussis, highest among infants, are pneumonia, seizures, encephalitis, and death. However, in many adolescents and adults, symptoms may be atypical, which makes the disease difficult to diagnose.

With the licensure of the whole cellular pertussis vaccine (DTP) vaccine in the late 1940s, the number of cases decreased substantially (100x). However, since the early 1990s the incidence of pertussis has increased. This increase may be attributable to improved diagnostic techniques, increased recognition, waning immunity, or a combination of these. Because of the globally increasing incidence of pertussis, particularly in adolescents and adults, at least 37 countries (not including the United States) have recommendations for a booster dose of acellular pertussis vaccine for adolescents and adults.

In Massachusetts, pertussis has been a reportable diseases since 1910. Surveillance for pertussis in Massachusetts includes a passive laboratory-based and active and passive epidemiologic surveillance systems. After the licensure of the vaccine in 1949, cases dropped precipitously. However, since the late 1980s, the number of reported pertussis cases has increased, and Massachusetts has one of the highest incidences in the country. From 1989 to 2002, the incidence in infants and children 1–10 years of age has remained stable. However, the incidence in adolescents has doubled, and the incidence in adults has increased fourfold. When assessing outbreaks, >80% have been associated with schools.

National and Massachusetts data have documented an increase incidence in adults and adolescents since the beginning of the 1990s, with substantial death associated with disease. With the availability of information, the Health and Human Services Advisory Committee on Immunization will need to decide whether to recommend a booster dose for the adolescent and adult population.

## Vaccine for Older Women

Women >65 years have a decreased T cell and B cell response, which results in diminished reactivity to antigens and a decreasing response to hepatitis B, pneumococcal, and influenza vaccines. Influenza and pneumococcal diseases cause substantial illness and death in persons >65 years. For influenza, an estimated 114,000 hospitalizations and 51,000 deaths occur annually, and 80%–90% of complications occur in persons >65 years. With the use of a trivalent vaccine in the elderly nursing home population, 80% of deaths and 30%–40% of confirmed influenza illnesses are prevented. Vaccination rates for influenza are 67% in persons >65 years; however, the difference between races is substantial, with a rate of 70% in non-Hispanic whites, 47% in Hispanics, and 52% in non-Hispanic blacks.

For invasive pneumococcal diseases, data collected in the Active Bacterial Core Surveillance in 1998 documented that the incidence of invasive pneumococcal disease is highest in children <1 year and persons >50 years. Invasive pneumococcal diseases include bacteremia, meningitis, and pneumonia. Annually >50,000 cases of bacteremia occur, with higher rates among the elderly, who have 60% of fatal cases. For pneumococcal pneumonia, an estimated 175,000 patients are hospitalized annually, and elderly patients have a higher death rate. Persons >65 years are recommended to receive the 23 valent polysaccharide vaccine. According to the Behavioral Risk Factor Surveillance Survey, coverage for persons >65 years is 66%; vaccination levels lower are in blacks (45%) and Hispanic persons (44%). Issues with pneumococcal vaccine include use of the conjugate vaccine for adults and frequency of revaccination. Several other important vaccines include tetanus and diphtheria and travel vaccines. Other vaccines for the elderly include varicella vaccine to prevent shingles.

